# Assessing hospital performance indicators. What dimensions? Evidence from an umbrella review

**DOI:** 10.1186/s12913-020-05879-y

**Published:** 2020-11-12

**Authors:** Elettra Carini, Irene Gabutti, Emanuela Maria Frisicale, Andrea Di Pilla, Angelo Maria Pezzullo, Chiara de Waure, Americo Cicchetti, Stefania Boccia, Maria Lucia Specchia

**Affiliations:** 1 Fondazione Policlinico Universitario A. Gemelli IRCCS, Rome, Italy; 2 Dipartimento di Scienze della Vita e Sanità Pubblica, Università Cattolica del Sacro Cuore, Largo F. Vito 1, 00168 Rome, Italy; 3 Alta Scuola di Economia e Management dei Sistemi Sanitari (ALTEMS), Università Cattolica del Sacro Cuore, Rome, Italy; 4 Local Health Authority, ASL Roma 1, Rome, Italy; 5 Dipartimento di Medicina Sperimentale, Università di Perugia, Perugia, Italy

**Keywords:** Hospital performance, Performance dimensions, Performance measurement, Hospital evaluation, Quality, Indicator

## Abstract

**Background:**

Patients’ increasing needs and expectations require an overall assessment of hospital performance. Several international agencies have defined performance indicators sets but there exists no unanimous classification. The Impact HTA Horizon2020 Project wants to address this aspect, developing a toolkit of key indicators to measure hospital performance. The aim of this review is to identify and classify the dimensions of hospital performance indicators in order to develop a common language and identify a shared evidence-based way to frame and address performance assessment.

**Methods:**

Following the PRISMA statement, PubMed, Cochrane Library and Web of Science databases were queried to perform an umbrella review. Reviews focusing on hospital settings, published January 2000–June 2019 were considered. The quality of the studies selected was assessed using the AMSTAR2 tool.

**Results:**

Six reviews ranging 2002–2014 were included. The following dimensions were described in at least half of the studies: 6 studies classified efficiency (55 indicators analyzed); 5 studies classified effectiveness (13 indicators), patient centeredness (10 indicators) and safety (8 indicators); 3 studies responsive governance (2 indicators), staff orientation (10 indicators) and timeliness (4 indicators). Three reviews did not specify the indicators related to the dimensions listed, and one article gave a complete definition of the meaning of each dimension and of the related indicators.

**Conclusions:**

The research shows emphasis of the importance of patient centeredness, effectiveness, efficiency, and safety dimensions. Especially, greater attention is given to the dimensions of effectiveness and efficiency. Assessing the overall quality of clinical pathways is key in guaranteeing a truly effective and efficient system but, to date, there still exists a lack of awareness and proactivity in terms of measuring performance of nodes within networks. The effort of classifying and systematizing performance measurement techniques across hospitals is essential at the organizational, regional/national and possibly international levels to deliver top quality care to patients.

## Background

Addressing issues related to quality of care is one of the major concerns of healthcare systems [1]. It is a current topic on the agenda of policy makers at different levels and worldwide [2] because of a growing need to control costs and guarantee sustainability, reduce variability in healthcare delivery, ensure transparency and accountability, deliver effective, safe and person-centered care, improve patients’ clinical outcomes and their satisfaction [2, 4].

Scientific literature on the topic is wide and evidence is available on different aspects of quality of care. These lead to several perspectives such as, for example, area or level of care, type of organization, improvement strategies [2].. Despite the broad literature, disagreement persists on what the expression “quality of care” comprehends and there is no unanimous understanding of the term [2]. The definition of Donabedian, who defines quality as “the ability to achieve desirable objectives using legitimate means”, is perhaps the one that better describes the concept in healthcare [2]. Also, according to Donabedian, quality of care is referred to the whole process of care, where the goal is to maximize general patient welfare and health outcomes [2].. The internationally accepted definition of the Institute of Medicine also recalls health outcomes and gives importance to evidence and professional knowledge [5]. Moreover, this last definition and the one formulated by World Health Organization (WHO) in 2006 [5] encompass other characteristics of care delivery. Healthcare has to be safe, effective, patient-centered, timely, efficient, equitable, acceptable and accessible. Among these features, safety, effectiveness and patient-centeredness/responsiveness can be considered universally as core dimensions while others can be viewed as subdimensions [2]. This distinction is based on the framework (Health Care Quality Indicator (HCQI) project) formulated by the Organization for Economic Co-operation and Development (OECD) in 2006 with the intent to lead the development of indicators in order to compare quality at the international level [2]. Indicators are indirect measures that provide information about the dimensions of quality of care [2]. Measurement is important to assess quality and implement improvement actions in order to provide better healthcare and enhance health outcomes. The use of standardized indicators leads on one hand to a better assessment across all levels of healthcare, on the other, to the increase of transparency and trust by patients [5]. Quality is not a synonym of performance but it is an important component of healthcare systems’ performance. This latter concept is therefore wider than the one of quality and describes the extent to which health systems are able to reach their goals [2].

Monitoring healthcare providers’ performance is relevant worldwide, especially in settings such as hospitals, given their significant weight in terms of both health and economic effects. In 2003, WHO launched a project aimed at supporting hospitals in order to develop a framework for the assessment of their performance. The project – named PATH (Performance Assessment Tool for quality improvement in Hospital) – was aimed at identifying dimensions and indicators for assessing hospital performance [4]. However, despite the detection of a great number of hospital performance indicators, some gaps in their measurement as well as issues concerning the dimensions investigated still exist. For example, some dimensions are under-represented and some healthcare settings or clinical specialties are not well tracked [6].

This study is part of the IMPACT HTA (Improved Methods and ACtionable Tools for enhancing HTA) project. IMPACT HTA is a Horizon 2020 research project aimed at studying variations in costs and health outcomes and at improving economic evaluation regarding HTA (Health Technology Assessment) and health system performance measurement through the integration of clinical and economic data from different sources. The measurement of hospital performance and its link with organizational models is one of the core aspects of the project. Within the latter, the authors of this paper dealt with the identification and classification of the dimensions of hospital performance indicators, according to scientific evidence, with the double aim of synthesizing the available knowledge on the topic in a comprehensive manner and providing the basis for a possible common way of measuring hospital performance.

## Methods

An umbrella review was performed with the aim of aggregating findings from reviews available on the topic. Indeed, an umbrella review allows to aggregate findings from reviews, making it possible to reassemble the fragmented knowledge concerning a specific topic. In other words, it provides a synthesis of the evidence described in several reviews that has been conducted on the same topic [7, 8].

### Search strategy

To perform an umbrella review, PubMed, Cochrane Library, and Web of Science databases were queried through the following search string: ((((Indicator* OR standard OR benchmark*) AND (project* OR program OR tool* OR set OR model)) AND ((outcome OR output) OR (quality OR effectiveness OR safety OR appropriateness OR efficiency OR “patient satisfaction” OR efficacy OR equity OR accountability OR “patient centredness” OR “patient centeredness” OR “staff orientation” OR “staff satisfaction” OR access*))) AND (measure* OR assess* OR evaluate OR implement*)) AND (performance AND “hospital*”). The investigation was conducted in June 2019 and the articles retrieved were screened according to the inclusion/exclusion criteria previously stated and listed in the following paragraph.

### Inclusion/exclusion criteria

The selection of the articles was conducted referring to previously defined inclusion and exclusion criteria. Only review articles focused on indicators of hospital performance, written in English or Italian, ranging from January 1st, 2000 to June 30th, 2019 and available in full text were considered. The included reviews had to classify the dimensions of hospital performance; articles analyzing only one dimension were maintained. Articles examining single indicators or lacking a classification in dimensions or reporting a classification only related to a single clinical setting or specialty were excluded.

### Data synthesis

The selection of the articles followed the PRISMA statement [9]. Two researchers screened independently the results by title and abstract and finally by full text. Eventual disagreements were discussed between the researchers in order to find a solution and in case of persistent discordance a third researcher was involved in the decision. To synthetize the selected articles, a table was designed and the following information were extracted: authors, country, year of publication, title, objective, results, dimension/subdimension, indicators, definition of each indicator. A qualitative synthesis was performed to analyze the data. A quantitative synthesis was not carried out due to the heterogeneity of the data retrieved.

### Quality appraisal

The quality of the studies selected was assessed using AMSTAR2 (A MeaSurement Tool to Assess systematic Reviews). AMSTAR2 allows to evaluate different aspects of reviews in order to define the quality of the studies. It enables researchers to carry out rapid and reproducible assessments to evaluate the published version of a review. The tool is composed of 16 domains in the form of questions that can be answered with ‘yes’; ‘partial yes’ or ‘no’. Seven out of 16 are the domains which can critically affect the validity of the review (in particular, domains 2, 4, 7, 9, 11, 13, and 15). The review is finally rated with a high, moderate, low or critically low overall confidence [10].

### Reporting

The whole document has been produced bearing in mind the PRISMA statement indications [9].

## Results

**Fig. 1:**
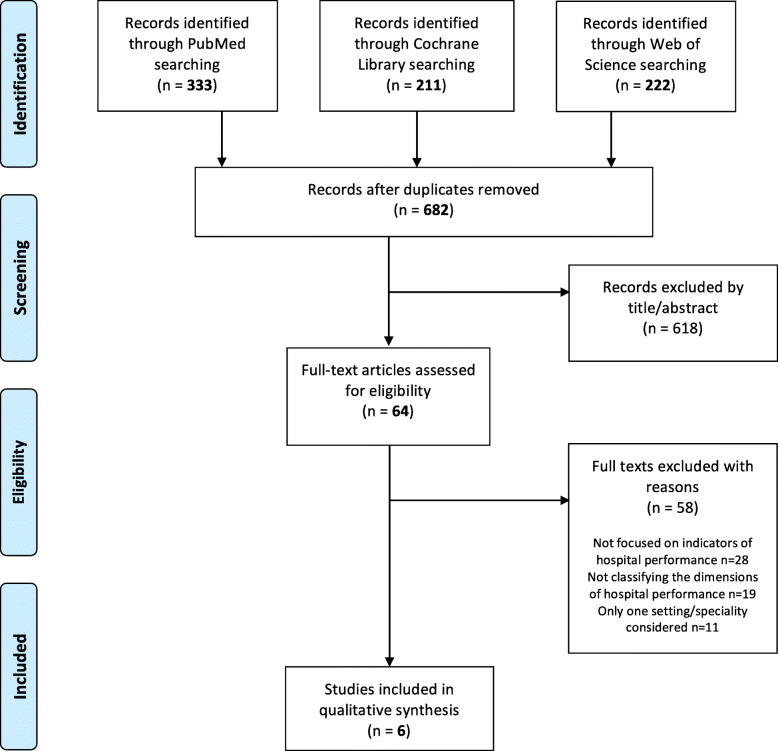
Flowchart depicting literature search and study selection

From the search, 222 records were identified in Web of Science, 333 in PubMed and 211 in Cochrane Library (Fig. 1). Duplicates removing was conducted through Mendeley software and a total number of 682 records was subsequently screened. A spreadsheet was compiled and, carefully reading the titles, 64 studies were assessed for eligibility. Six reviews were finally included in the qualitative synthesis.

The six reviews retrieved range from 2002 to 2014. Their main characteristics and results are summarized in Table 1. All the studies report a classification of the indicators in diverse dimensions but not all of them specify the indicators included in each. The ones only describing the dimensions are three [1, 3, 6]. The review by Veillard et al. gives a deeper insight providing a definition of the dimensions and a precise definition of each indicator described [4]. A subclassification of the dimensions is proposed by two reviews: Ganjour et al. group the indicators according to the disease or intervention they refer to [11], while Veillard et al. specify each dimension according to specific and detailed characteristics [4].

**Table 1:** Characteristics of the reviews included

1st author, Year, Country	Databases Date range	Title	Objective	Main results
Gandjour A, 2002 [11] (Germany)	PubMed Up to July 2000	An Evidence-Based Evaluation of Quality and Efficiency Indicators.	To identify and appraise quality and efficiency indicators relevant to hospitals or physicians’ practices.	Seven structural indicators and 34 process indicators were identified and appraised. The set of performance indicators could serve as a state-of-the-art system of measurement for governments and organizations evaluating the quality and efficiency of healthcare.
Veillard J, 2005 [4] (Spain, Canada, The Netherlands, USA)	PubMed Web Of Science 2003–2005	A performance assessment framework for hospitals: the WHO regional office for Europe PATH project.	To describe the first step of PATH project: to develop an overall framework for hospital performance assessment.	Six dimensions were identified: clinical effectiveness (3 subdimensions: appropriateness of care, conformity of processes of care, n outcomes of care and safety processes for a total number of 7 indicators), safety (2 indicators), patient centeredness (5 indicators), production efficiency (3 subdimensions: appropriateness of services, productivity, use of capacity for a total of 4 indicators), staff orientation (3 subdimensions: perspective and recognition of individual needs, health promotion and safety initiatives, behavioral response for a total of 4 indicators) and responsive governance (2 subdimensions: system integration and continuity, public health orientation with 1 indicator each).
Groene O, 2008 [3] (Spain, Denmark)	PubMed Web Of Science 1995–2008	An international review of projects on hospital performance assessment.	To identify and compare current indicator projects, and raise questions regarding the impact of hospital performance assessment that should be pursued.	Eleven projects were included that appear to have adopted a common methodology for the design and selection of indicators; six dimensions were described: clinical effectiveness, staff orientation, responsive governance, safety, patient centeredness, efficiency.
Copnell B, 2009 [6] (Australia)	PubMed Web Of Science 1999–2009	Measuring the quality of hospital care: an inventory of indicators.	To identify and classify indicators currently in use to measure the quality of care provided by hospitals, and to identify gaps in current measurement.	383 discrete indicators were identified from 22 source organizations or projects. 27.2% were hospital-wide, 26.1% were related to surgical patients and 46.7% to non-surgical specialties, departments or diseases. Cardiothoracic surgery, cardiology and mental health were the specialties with greatest coverage, while nine clinical specialties had fewer than three specific indicators. Processes of care were measured by 54.0% of indicators and outcomes by 38.9%. Safety and effectiveness were the domains most frequently represented, with relatively few indicators measuring the other dimensions. The dimensions described were safety, effectiveness, efficiency, timeliness, patient-centeredness, equity.
Beyan OD, 2012 [1] (Turkey)	PubMed Web Of Science 2000–2012	A Knowledge Based Search Tool for Performance Measures in Health Care Systems.	To design a tool that simplifies the performance indicator search process and to provide most relevant indicators by employing knowledge based systems.	A multidimensional conceptual framework to identify features of performance measurement was designed. Through literature analysis, 4 main strata were found which defined the performance measurement studies: stakeholder, data, indicator and target levels. The dimensions described were acceptability, accessibility, appropriateness, care environment and amenities, continuity, competence or capability, effectiveness, improving health or clinical focus, expenditure or cost, efficiency, equity, governance, patient-centeredness, safety, sustainability, timeliness, utilization.
Simou E, 2014 [12] Greece	PubMed Web Of Science 1980–2010	Developing a national framework of quality indicators for public hospitals.	To describe the development of a preliminary set of quality indicators for public Greek National Health System hospitals.	Twenty relevant projects and their 1698 indicators were selected through a literature search, and after the consensus panel process, a list of 67 indicators were selected to be implemented for the assessment of the public hospitals categorized in the following dimensions: effectiveness (6 indicators), safety (6 indicators), patient-centeredness (5 indicators), staff orientation (6 indicators), efficiency (10 indicators), utilization (5 indicators), timeliness (4 indicators), and resources and capacity (25 indicators).

### The dimensions of performance indicators

**Fig. 2:**
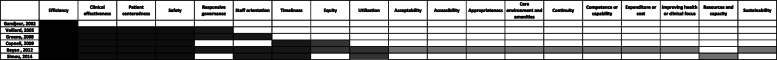
The dimensions of hospital performance

The dimensions in which the indicators of hospital performance have been classified are (Fig. 2): efficiency (6 studies; 100%) [1, 3, 4, 6, 11, 12], clinical effectiveness (5 studies; 83%) [1, 3, 4, 6, 12], patient-centeredness (5 studies; 83%) [1, 3, 4, 6, 12], safety (5 studies; 83%) [1, 3, 4, 6, 12], responsive governance (3 studies; 50%) [1, 3, 4], staff orientation (3 studies; 50%) [1, 3, 12], timeliness (3 studies; 50%) [1, 6, 12], equity (2 studies; 33%) [1, 6], utilization (2 studies; 33%) [1, 12]. All the other dimensions were described by only 1 (17%) article: acceptability [1], accessibility [1], appropriateness [1], care environment and amenities [1], continuity [1], competence or capability [1], expenditure or cost [1], improving health or clinical focus [1], resources and capacity [12], and sustainability [1]. The main findings of this work are summarized in the following paragraphs, while detailed information is presented in Table 2.

**Table 2:** Dimensions, sub-dimensions and indicators reported by the 6 reviews

1st AUTHOR, YEAR	DIMENSION	SUBDIMENSION^a^	INDICATORS^a^
Gandjour A, 2002 [8]	Process quality/efficiency		*Acute miocardial infarction*
			Reperfusion using either thrombolytics during the first 12 h of pain onset or primary PTCA
			Use of aspirin during hospitalization
			Use of lidocaine during hospitalization
			Use of a β-blocker (acebutolol, metoprolol, propranolol, timolol) during hospitalization
			Use of an ACE inhibitor during hospitalization
			Use of statin during hospitalization for total cholesterol levels ≥6 mmol/L
			*Acute stroke*
			Chest x-ray on admission
			Brain imaging
			Thrombolytic therapy within 0 to 3 h of onset
			Aspirin or clopidogrel or dipyridamole or ticlopidine for ischemic stroke
			Anticoagulants, calcium antagonists, change in blood pressure medication, corticosteroids, gangliosides, glycerol, hemodilution, heparinoids, low–molecular-weight heparins, piracetam, standard unfractionated heparin
			*Coronary artery bypass graft*
			Appropriateness of indication
			*Percutaneous transluminal coronary angioplasty*
			Appropriateness of indication
			*Carotid endarterectomy*
			Appropriateness of indication
			*Acute lower back pain*
			Bed rest
			Exercise therapy
			Immediate x-ray
			Other diagnostic procedures within the first 4 weeks of symptoms
			Referral to another provider without specific request
			*Major depressive disorders (acute episode)*
			Use of DSM-IV or ICD-10 criteria for diagnosis
			Use of newer or older antidepressants or problem-solving treatment or interpersonal psychotherapy or nondirective counseling or cognitive behavior therapy (conducted at own office or by referral)
			Duration of drug prescription (minimum of 24 weeks)
			*Type 2 diabetes mellitus*
			Test HbA1c once every 6 months
			Biennial testing of fasting serum total cholesterol, triglycerides, HDL cholesterol, and LDL cholesterol
			Annual urine test for (micro-) albuminuria
			Annual testing of blood pressure
			Annual foot examination including testing for pain, touch, cold, vibration, ankle reflexes, and pressure
			Annual foot examination including foot structure and biomechanics, vascular status, and skin integrity
			Biennial eye examination by an ophthalmologist or optometrist
			Patient education
		Screening programs	Biennial hemoccult screening for colorectal cancer at age ≥ 50 years
			Sigmoidoscopy for colorectal cancer every 10 years at age ≥ 50 years
			Measurement of the prostate-specific antigen (prostate cancer)
			Papanicolaou smear at least every 5 years for women who are sexually active and between 30 to 60 years old (cervical cancer)
	Structural quality/efficiency		Implementation of evidence-based clinical practice guidelines
			(hospitals)
			(unstable angina)
			Implementation of evidence-based clinical practice guidelines
			(physicians’ offices)
			(cancer pain)
			Computer alert system to prevent injury from adverse drug events (hospitals)
		Antibiotic improvement intervention	Structured antibiotic order forms
			Academic detailing
Veillard J, 2005 [4]	Clinical effectiveness and safety	Appropriateness of care	Cesarean section delivery
		Conformity of processes of care	Prophylactic antibiotic use for tracers: results of audit of appropriateness
		Outcomes of care and safety processes	Mortality for selected tracer conditions and procedures
			Readmission for selected tracer conditions and procedures
			Admission after day surgery for selected tracer procedures
			Return to higher level of care (e.g. from acute to intensive care) for selected tracer conditions and procedures within 48 h
			Sentinel event
	Safety	Staff safety	Percutaneous injuries
			Staff excessive weekly working time
	Patient centredness	Client orientation, respect for patients	Average score on overall perception/satisfaction items in patient surveys
			Average score on interpersonal aspect items in patient surveys
			Last minute cancelled surgery
			Average score on information and empowerment items in patient surveys
			Average score on continuity of care items in patient surveys
	Responsive governance	System integration and continuity	Average score on perceived continuity items in patient surveys
		Public health orientation	Breastfeeding at discharge
	Staff orientation	Perspective and recognition of individual needs	Training expenditures
		Health promotion and safety	Expenditures on health promotion activities
		Behavioural responses	Absenteeism: short- term absenteeism
			Absenteeism: long- term absenteeism
	Efficiency	Appropriateness of services	Day surgery, for selected tracer procedures
		Productivity	Length of stay for selected tracers
		Use of capacity	Inventory in stock, for pharmaceuticals
			Intensity of surgical theatre use
Groene I, 2008 [3]	Clinical Effectiveness Staff orientation Responsive governance Safety Patient Centeredness Efficiency		
Copnell B, 2009 [6]	Safety		
	Effectiveness		
	Efficiency		
	Timeliness		
	Patient-centeredness		
	Equity		
Beyan OD, 2012 [1]		Acceptability	
		Accessibility	
		Appropriateness	
		Care environment and amenities	
		Continuity	
		Competence or capability	
		Effectiveness	
		Improving health or clinical focus	
		Expenditure or cost	
		Efficiency	
		Equity	
		Governance	
		Patient centeredness or patient focus or responsiveness	
		Safety	
		Sustainability	
		Timeliness	
		Utilization	
Simou E, 2014 [9]	Effectiveness		Inpatient mortality from selected causes (AMI, stroke, pneumonia, etc.)
			Readmission rate for selected causes
			Unscheduled readmission to ICU
			Perioperative mortality
			Perinatal mortality due to complications
			Cancer patients successfully surviving surgery/chemotherapy/transplant
	Safety		In-hospital avoidable VTE
			Hospital-acquired infections (VAP, urinary catheter associated UTI, central line associated blood stream, surgical site, infections in neonates)
			Medical errors per sector (post- surgery, improper treatment, iatrogenic)
			Obstetric trauma
			Staff injury
			Staff needle puncture incidents
	Patient centeredness		Patient feedback management
			Pain control
			Satisfaction from personnel
			Explanation of procedures, treatment and discharge information
			Satisfaction from hospital environment (cleanliness, quietness, privacy)
	Staff orientation		Staff burnout
			Staff absenteeism
			Staff working overtime
			Satisfaction from working environment
			Clearly defined responsibilities in staff
			Continuous education for health professionals
	Efficiency		Length of stay
			ICU length of stay
			Hospital bed coverage
			Admission/discharge rate
			Cost of inpatient services per patient day
			Exams ordered at the ER, per patient
			Laparoscopic/open surgery rate
			Single-day stay for selected surgeries
			Caesarian section rate
			Surgery postponed or cancelled
	Utilization		Patients visiting the ER department
			Admissions for acute conditions
			Usage of equipment/facilities
			Usage of laboratory exams
			Surgical Theater use
	Timeliness		Time needed for initial clinical examination at the ER after arrival
			Time needed for admission after arrival at the ER
			Time needed for selective surgical treatment
			Patients leaving without being examined
	Resources and capacity		Permanent personnel (per discipline)
			Detached personnel (per discipline)
			Temporary personnel (per discipline)
			Personnel educational level (per discipline)
			Intra-sector nurses to physicians ratio
			Computers for the personnel
			Computers with Internet access
			Computers with modern applications
			Use of electronic medical records
			Hospitals having a webpage
			Telephone center
			Surgical theaters
			Beds per sector
			Beds per room
			Short-term stay beds
			Space for patient baggage
			Toilet in patients’ rooms
			Intra-communication facilities in patients’ rooms
			Oxygen facilities in patients’ rooms
			Air-conditioning facilities in patients’ rooms
			Telephone facilities in wards
			Imaging facilities (radiography, ultrasound, CT, MRI, etc.)
			ICU and HCU unit(s)
			Hemodialysis facilities
			Management of hospital waste

^a^The blank sections of the columns “subdimension” and “indicators” are due to a lack of those information in the corresponding review

### Efficiency

Efficiency has been explored by all the reviews included [1, 3, 4, 6, 11, 12]. It can be defined as the optimal allocation of available healthcare resources that maximize health outcomes for society [13]. Veillard et al. similarly refer to efficiency as hospital optimal use of inputs to yield maximal outputs, given the available resources [4]. The review by Gandjour et al. considers efficiency in terms of process (referring to specific clinical conditions) and structure [11]. Veillard et al. also provide a subclassification of the dimension, describing efficiency in terms of appropriateness of services, productivity, and use of capacity [4].

### Clinical effectiveness

According to Veillard et al., clinical effectiveness is the appropriateness and competence which allows to deliver clinical care and services with the maximum benefit for all patients [4]. This study sub-classifies this dimension in appropriateness of care, conformity of processes of care, and outcomes of care and safety processes [4]. Simou et al., take into account mortality rates, readmissions, and survival [12].

### Patient-centeredness

As reported by Veillard et al., this dimension concerns a set of indicators which pay attention to patients’ and families’ orientations. The main aim is to evaluate whether patients are placed at the center of care and service delivery [4]. Simou et al. provide patient centeredness indicators in terms of patients’ feedback [12].

### Safety

Safety refers both to patients and professionals in terms of the ability to avoid, prevent and reduce harmful interventions or risks for them and for the environment [4, 12].

### Responsive governance

Another aspect analyzed by three out of the six reviews is responsive governance [1, 3, 4]. Veillard et al. provide a definition of responsive governance which is described as the “degree of responsiveness to community needs, to ensure care continuity and coordination, to promote health and provide care to all citizens” and subclassify the related indicators into system integration and continuity and public health orientation [4]. The other two studies do not specify the set of indicators related to this dimension [1, 3].

### Staff orientation

Staff orientation is reported by three studies [1, 3, 12]. This dimension is considered by Veillard et al. in terms of recognition of individual needs, health promotion and safety initiatives, behavioral responses [4]. Simou et al. address the issue through absenteeism, working environment satisfaction, overtime working, burnout and continuous education [12].

### Timeliness

Timeliness [1, 6, 12] is assessed in terms of indicators only by Simou et al. and it refers to the time needed to be addressed to specific treatments [12].

### Other dimensions

The other dimensions described are only addressed by one or two studies or only cited without specifying the related indicators. Just utilization and resource and capacity are described by Simou et al. [12]. The utilization dimension refers to the use of facilities and equipment, while the resources and capacity one analyzes the amount of personnel and equipment used.

## Discussion

The present study aimed to identify and classify the dimensions of hospital performance indicators in order to make a contribution to the development of a common language and to identify a common evidence-based way to frame and address performance assessment. It is widely recognized that assessing hospital performance takes into account multiple aspects and dimensions, given the organizational and procedural complexity of such entities and the high number of stakeholders involved in their activities, likely to set different priorities as well as actual meanings to the term “performance” [14].

Therefore, it is still difficult to assess the performance of hospitals concretely. The task is even more challenging if we consider the differences in the main characteristics of hospitals (e.g. dimension, ownership, degree of clinical focus, geographical location).

This review contributes in shedding light on the most common ‘clusters’ of hospital performance indicators. As predictable [15], the dimensions of effectiveness and efficiency are highly emphasized and these appear to be the most represented dimensions from our review. Alongside them, the dimensions of safety and patient-centeredness are also well represented in the included reviews. Interestingly, a number of further dimensions of performance emerges, held into account in a non-systematic way or only sporadically. This may provide an interesting picture of how performance is intended and understood concretely in our healthcare systems.

Clearly, the *objective* of healthcare organizations is to produce health, so clinical effectiveness is probably its most direct proxy. Effectiveness, however, must be consistent with efficiency, in the not less relevant challenge of ensuring organizational sustainability in the long run. The lively debate on conflicts emerging between clinical professionals and managerial teams testifies the challenging need of combining the perspectives of roles that tend to focus on different priorities [16].

Performing in terms of safety is strictly connected to clinical effectiveness. The capability of a hospital to preserve patients’ good health is the other side of the coin in assuring clinical quality. Clearly, a hospital should not only intervene to improve patients’ health, but it must do so in such a way as to avoid exposing them to potential sources of personal harm. Moreover, safety is also referred to staff, which is frequently directly exposed to numerous risks and requires a structured and solid organizational apparatus to preserve its health [17]. The dimension of patient-centeredness may be interpreted in a strictly interconnected way with that of responsive governance [18]. If, on the one hand, patient-centeredness may assume a ‘double face’, covering both the dimension of patient satisfaction as well as that of continuity of care, on the other, responsive governance seems related to the capability of the hospitals to monitor performance in an integrated manner (within its units and throughout different settings). The last topic surely needs an increasingly relevant focus in the years to come [19].

Indeed, in accordance with epidemiological patterns of developed countries, with ageing populations and the spread of multi-pathological chronic conditions, it is crucial to shift the conception of performance from a setting-oriented to a multi-setting oriented approach. Hospitals are by now *one* of the steps in patients’ clinical pathways and it is misleading to assess their contribution to their final status of health in a completely isolated way [20]. Indeed, although it is important to isolate the effects of a specific setting from the others, it makes little sense to think of performance as something that can be obtained without structured interconnections with the other settings of the system. In this sense, all the efforts in assessing the *overall* quality of clinical pathways are key in guaranteeing a truly effective and efficient system. As noted, however, only a limited percentage of the scientific evidence assessed in this study takes into account this perspective. This may suggest that, to date, there still exists an important lack of awareness and proactivity in terms of measuring performance of nodes within networks rather than of isolated monads.

Furthermore, the effects of possibly non-systematized ways of measuring performance are likely to exert heavy repercussions in a number of ways. These may include lack of transparent information to patients, career choices of professionals, access to public and private funds [21].

It is therefore clear that the effort of classifying and systematizing performance measurement techniques across hospitals is key at the organizational, regional/national and possibly international levels.

At the organizational level, the way in which performance is assessed is likely to have strong and direct effects on internal organizational and managerial equilibria, as well as on the overall strategy of the hospital. Depending on the choice of ‘key’ performance dimensions, some units or directorates are likely to be held more or less performing. This, in turn, can affect internal equilibria due to, for example, prestige and allocation of resources [22].

At the regional/national level this sort of assessment may have crucial effects on the access to resources. Not only in terms of monetary remunerations (due to patients’ choice of being assisted in a certain hospital or to, as is the case in some healthcare systems, the decision of regions to finance more or less large amounts of clinical activities within that hospital), but also in terms of their appeal to professionals and industries of health technologies [23].

At the international level, a common ground to assess performance would allow an indirect assessment of different healthcare systems. These are highly differentiated across countries and benchmarking efforts are frequently hindered by the lack of comparable data [24].

In this scenario, it is crucial to provide the basis for a possible common way of measuring hospital performance. This analysis provides a solid first step in this direction.

The study presents some limitations, hereby presented. No quantitative synthesis of the studies was conducted as there was high heterogeneity among the reviews included and therefore among their results. The dimensions were described by all the studies but only half of them specified the respective indicators and the list presented is not comprehensive of all the possible indicators. The overall quality of the review was assessed to be ‘critically low’ as the reviews did not meet the quality criteria stated by the AMSTAR2 tool, even though it has to be pointed out that this tool does not fit completely the needs of a review centered on organizational/management topics. Despite these limitations, the proposed review represents, so far, the first attempt to synthetize the available knowledge on the topic in a comprehensive manner and with a strict methodology. The inclusion of only reviews was aimed at providing stronger evidence.

### Quality assessment

All the included reviews, assessed for quality through AMSTAR2 tool, have been rated as critically low (Annex 1). All the reviews did not meet 11 out of 16 domains of the AMSTAR2 scale. Five out of those 11 domains are critical and are referred to: (7) justification for the exclusion of studies which were not included in the review; (9) assessment of the risk of bias (RoB) in individual studies which were included in the review; (11) use of appropriate methods for statistical combination of results if meta-analysis was performed; (13) taking into account RoB in individual studies when interpreting/discussing the results of the review; (15) adequate investigation of publication bias if quantitative synthesis was performed and discussion of its likely impact on the results of the review.

## Conclusions

The assessment of the overall quality of clinical pathways is key in guaranteeing a truly effective and efficient system. The effort of a deeper understanding of what quality really means and which are the related dimensions is not new and in 2006 a framework aimed at the development of indicators to compare quality at the international level was settled by OECD [2]. Quality is made of different dimensions [2] thus, measuring quality means measuring those dimensions through the use of specific indicators. According to OECD, some dimensions, such as safety, effectiveness and patient-centeredness/responsiveness, can be considered universally as core dimensions when dealing with quality in healthcare [2]. Nevertheless, although some clusters of indicators may appear rather intuitive and universally monitored (although – even here – it should be assessed what is actually measured within each cluster), some clusters appear incredibly subject to interpretations or even overlooked. Therefore, the effort of clustering indicators into shared categories is fundamental to guarantee a systematic, reproducible, comparable, and universally shared evaluation but it also sheds light on the existing gaps concerning this evaluation and systematization. Further studies should provide guidance in covering this gap, as well as in highlighting how all the less frequently assessed dimensions of performance may be integrated within overall hospital assessments.

## Data Availability

All data generated or analysed during this study are included in this published article and its supplementary information files.

## Abbreviations

AMSTAR2: A MeaSurement Tool to Assess systematic Reviews
HCQI: Health Care Quality Indicator
HTA: Health Technology Assessment
IMPACT HTA: Improved Methods and ACtionable Tools for enhancing HTA
OECD: Organization for Economic Co-operation and Development
PRISMA: Preferred Reporting Items for Systematic Reviews and Meta-Analyses
PATH: Performance Assessment Tool for quality improvement in Hospital
RoB: Risk of Bias
WHO: World Health Organization
